# Vaccinia Virus: Mechanisms Supporting Immune Evasion and Successful Long-Term Protective Immunity

**DOI:** 10.3390/v16060870

**Published:** 2024-05-29

**Authors:** Joy Hsu, Suyon Kim, Niroshana Anandasabapathy

**Affiliations:** 1 Weill Cornell Graduate School of Medical Sciences, New York, NY 10065, USA; 2 Department of Dermatology, Weill Cornell Medicine, New York, NY 10021, USA; 3 Department of Microbiology and Immunology, Weill Cornell Medicine, New York, NY 10021, USA; 4 Meyer Cancer Center, Weill Cornell Medicine, New York, NY 10021, USA; 5 Englander Institute of Precision Medicine, Weill Cornell Medicine, New York, NY 10021, USA; 6 Renaissance School of Medicine, Stony Brook University, Stony Brook, NY 11794, USA; suyon.kim@stonybrookmedicine.edu

**Keywords:** vaccinia, adaptive immunity, skin, dendritic cell, T cell

## Abstract

Vaccinia virus is the most successful vaccine in human history and functions as a protective vaccine against smallpox and monkeypox, highlighting the importance of ongoing research into vaccinia due to its genetic similarity to other emergent poxviruses. Moreover, vaccinia’s ability to accommodate large genetic insertions makes it promising for vaccine development and potential therapeutic applications, such as oncolytic agents. Thus, understanding how superior immunity is generated by vaccinia is crucial for designing other effective and safe vaccine strategies. During vaccinia inoculation by scarification, the skin serves as a primary site for the virus–host interaction, with various cell types playing distinct roles. During this process, hematopoietic cells undergo abortive infections, while non-hematopoietic cells support the full viral life cycle. This differential permissiveness to viral replication influences subsequent innate and adaptive immune responses. Dendritic cells (DCs), key immune sentinels in peripheral tissues such as skin, are pivotal in generating T cell memory during vaccinia immunization. DCs residing in the skin capture viral antigens and migrate to the draining lymph nodes (dLN), where they undergo maturation and present processed antigens to T cells. Notably, CD8+ T cells are particularly significant in viral clearance and the establishment of long-term protective immunity. Here, we will discuss vaccinia virus, its continued relevance to public health, and viral strategies permissive to immune escape. We will also discuss key events and populations leading to long-term protective immunity and remaining key gaps.

## 1. Introduction

### 1.1. The Continued Relevance of Vaccinia Immunization: The Most Successful Vaccine in Human History

Between 1875 and 1975, Variola virus (smallpox) claimed the lives of an estimated 300–500 million individuals until its eradication in 1977 via global vaccination through the administration of vaccinia (VACV) by skin scarification (VACV_SS_) [1,2]. Given the 20–45% mortality rate of smallpox, vaccinia immunization is recognized as the most successful vaccination campaign in human history [3,4].

Vaccinia, the smallpox (VARV) vaccine, has been utilized to provide cross-immunity to the monkeypox virus (MPV) after MPV outbreaks in the US in 2003 and more recently in 2022 [5,6]. The substantial genetic similarity between poxviruses, such as between MPV and VARV, highlights the continued importance of VACV research to combat re-emergent orthopox outbreaks with potential implications for public health. Despite the significantly milder clinical presentation of monkeypox relative to smallpox, there is a notable 96.3% genetic correspondence between the central genomic regions of the monkeypox and that of the smallpox viral genome, including regions encoding structural proteins and essential enzymes [7]. Areas of future study include understanding the mechanisms supporting long-term protective immunity to VACV for designing safer and scalable strategies against orthopoxviruses, as well as understanding foundational VACV pathogenesis and immune evasion mechanisms. 

Currently, a live single VACV strain, ACAM2000™, protects the military from smallpox and the public from monkeypox. This second-generation smallpox vaccine was developed and implemented as a replacement for Dryvax^®^, a composite of VACV strains of varying virulence [8]. ACAM-2000™, like its predecessor Dryvax^®^, is live, replicating VACV, given by scarification with similar or superior take rates in vaccinia-naïve subjects, and plaque-reduction neutralization test (PRNT) antibody titers in vaccinia-experienced subjects [9,10]. Live viruses such as ACAM-2000™ pose significant systemic risks: frequent myocarditis and pericarditis, blindness from autoinoculation of the eye, and the infection of broken skin (e.g., eczema—10% lifetime incidence) can cause a fatal varicelliform skin eruption (eczema vaccinatum) with 7% mortality, and progressive vaccinia infection (in immunocompromised individuals) due to uncontrolled viral replication [11,12]. Additionally, the live virus cannot be used in pregnant or immunocompromised persons. Consequently, ACAM-2000™ use is avoided in populations at higher risk for HIV, including the current non-African cases of MPV and groups where the spread of MPV may be more pronounced, such as in sex workers [13]. Hence, the need for safer vaccines remains. 

To eliminate smallpox more safely, several replicative vaccinia strains were created worldwide. These include Western Reserve (WR), Copenhagen, Wyeth, Lister, NYCBH (New York City Board of Health), and Tiantan strains [14]. The WR VACV strain was derived by passaging the NYCBH (ACAM-2000™) strain of VACV through the brains of mice. Notably, WR-VACV is not a human vaccine strain and, instead, is used extensively in in vivo mouse studies [15]. Compared to Dryvax^®^, WR-VACV grows to 6- to 20-fold higher titers in the skin of mice [16]. To enhance safety, researchers attenuated several strains through successive passages. The continuous passaging of the chorioallantois vaccinia virus strain Ankara using chick embryo fibroblasts (CEFs) led to an attenuated and replication-deficient strain in mammalian cells called modified Vaccinia Ankara virus (MVA), which had lost 15% of the VACV viral genome [14,17]. 

Now deployed for monkeypox vaccination and military use, Jynneos^TM^ is a live, replication-restricted MVA strain delivered subcutaneously (s.c.) and was approved in 2022 to be delivered intradermally (i.d.) without scar and, consequently, does not elicit skin reactions at the inoculation site [18,19]. As a vaccine for smallpox, MVA has fewer significant adverse events with no cases of myopericarditis, is unlikely to spread to other sites or people due to the lack of replication in human cells, and is, therefore, a safe vaccination option for pregnant or immunocompromised patients [20]. Recently, Jynneos^TM^ was downgraded for efficacy as a vaccine for monkeypox due to a case–control study of 2193 case patients and 8319 control patients who had either received one or two doses of Jynneos^TM^ [19]. The study found that the estimated adjusted vaccine effectiveness for two-dose vaccination was 66% (95% confidence interval (CI), 47.4 to 78.1), and one-dose vaccination had an estimated adjusted vaccine effectiveness of 35.8% (95% CI, 22.1 to 47.1) [19]. Replication or administration by scarification may be variables distinguishing efficacy given the higher efficacy of ACAM-2000™ over Jynneos^TM^ in stimulating an immune response in individuals with a healthy immune system; thus, it remains essential to investigate the mechanisms of successful vaccine strategies [19,21]. 

VACV, in its recombinant form, exhibits significant promise for vaccine development due to its capacity for large insertions, stability and safety, and infectious and immunogenic properties. Recombination between VACV genomes and genetic material within infected cells can incorporate large foreign DNA sequences into the VACV genome. This holds potential for creating VACV-based vectors targeting viral infections, Additionally, there is emerging interest in the potential of VACV as an oncolytic agent for future therapeutic applications [14]. Lastly, understanding the success of vaccinia-solicited immunity is critical to develop new vaccine strategies and adjuvants to mimic live viral infection in a safer context. This review will provide an overview of vaccinia immunity, with a focus on mechanisms of vaccinia immune evasion and immune licensing. 

### 1.2. What Is Vaccinia (VACV)?

Due to its absence of a natural host and distinctiveness from smallpox (VARV) and cowpox (CPXV) viruses, VACV lineage has been a topic of speculation. One proposed theory suggests a potential transformation from smallpox by successive arm-to-arm passages; however, the genomic sequencing of orthopox viruses has excluded VARV as the origin of VACV. More recent studies have posited, on the basis of genomic examination and the pattern of gene inactivation, that horsepox virus (HPSV) is the likely ancestor of several VACV strains that were passaged as vaccines [22]. Moreover, a genomic examination of HSPV revealed notable similarities to VACV and rabbitpox virus, although HSPV also showcases unique sequences distinct from VACV-like viruses. Overall, these findings emphasize the complexity and evolutionary origins of VACV and related viruses [23].

### 1.3. VACV Gene and Protein Expression

VACV has 200 viral genes that can be expressed early, intermediate, early/late, and late in the viral replication cycle, depending on whether the promoters upstream of the genes are early, intermediate, and late or a combination of early and late [24,25]. As VACV packages a complete virus-encoded transcription system, early mRNAs may be synthesized after entry into the cytoplasm and before DNA replication [24]. Early genes encode proteins that inhibit the innate and adaptive immune response, as well as DNA replication factors that regulate the expression of intermediate-class genes, including transcription factors (TFs), ensuring a coordinated progression of the viral replication cycle [26]. The synthesis of proteins and DNA is required to transcribe additional genes, which are separated into intermediate and late post-replicative groups, expressed after viral genome replication [27]. Similarly, intermediate genes encode TFs responsible for regulating late gene expression. Meanwhile, late genes largely encode structural proteins, including progeny virion proteins essential for viral RNA transcription and early gene transcription factors [27]. 

## 2. Vaccinia and Innate Immunity

During VACV immunization, the skin is a crucial site for virus–host interplay. In vaccinia vaccination, hematopoietic cells including T cells, Langerhans cells, and dermal dendritic cells (DCs) undergo abortive infections, while keratinocytes, dermal microvascular endothelial cells, and dermal fibroblasts were shown to support the full viral life cycle [28]. The restriction of viral replication to specific cell types thus affects the downstream innate and adaptive immune response. 

### 2.1. Tropism

Poxviruses in humans, including VACV, exhibit a strong preference for binding primary human antigen-presenting cells (APCs), and are capable of binding murine monocytes, macrophages, and dendritic cells (DCs) [29]. Upon cutaneous vaccinia virus (VACV) inoculation, T cells, Langerhans cells, and dermal DCs undergo abortive infection [28]. Both VACV and attenuated MVA are capable of infecting paracortical DCs, sinus-resident macrophages, and immature conventional dendritic cells (cDC1 and cDC2) proximal to lymph conduits [30]. Notably, the use of recombinant WR-VACV reporter viruses VACV-EGFP and vA5L-YFP to visualize viral binding and infection of ex vivo B cells reveals that susceptibility of primary human B cells to VACV is dependent upon B cell stage due to receptor expression, such as CXCR5, on the surface of B cells [31]. Specifically, memory B cells, critical for robust antibody-mediated immune response upon infection, are preferentially infected, whereas plasmablasts, plasma, transitional, and mature naïve B cells exhibit resistance to VACV infection [31]. Further, while VACV can productively infect activated B cells, VACV infection of ex vivo B cells is abortive [31]. Finally, a study of VACV zoonotic outbreaks in Brazil demonstrated reduced peripheral B counts in infected individuals compared to their uninfected counterparts, indicating the impact of VACV on the host B cell population [32]. 

Antigen-presenting cells (APCs) infected in vitro cannot express late viral proteins [33]; therefore, DCs cannot present and elicit immunity against late expression VACV proteins. Using early and late viral promoters to control the expression of model antigen β-galactosidase in various recombinant vaccinia viruses, only DCs infected with a recombinant VACV expressing β-galactosidase under early promoters could drive the antigen-specific CD8+ T cell response and protect against secondary challenge [33]. 

Conversely, VACV is replication-competent in non-hematopoietic cells, such as keratinocytes, dermal fibroblasts, and dermal microvascular endothelial cells [28]. In cultured human skin cells, VACV-infected epidermal and follicular keratinocytes facilitate low-level viral replication, whereas dermal fibroblasts and endothelial cells support robust viral replication [28]. VACV skin scarification results in uniform epidermal and follicular keratinocyte infection, which derives significantly elevated viral gene expression, and thus antigen availability, in contrast to subcutaneous delivery [34]. 

### 2.2. Lymphatic Drainage

Lymphatic vessels, though previously viewed as passive conduits, facilitate communication between the innate and adaptive immune systems by coordinating DC migration and the transport of soluble antigen to dLN [35]. Additionally, the immunization route has been shown to affect whether soluble antigens or DCs go on to drive an antigen-specific lymphocyte response.

Viral dissemination to the dLN depends on the route of immunization. After skin scarification, lymphatic clamping helps to limit rapid viral dissemination to distal and contralateral sites and the dLN. After VACV skin scarification (s.s.), but not VACV i.d., the lymphatic remodeling of inter-endothelial junctions occurs in a type I interferon-dependent manner, which lengthens endothelial capillary tips, sequesters lymph fluid, and prevents the spreading of viral antigen and infection [36]. In particular, lymphatic “zippering”, defined as dermal lymphatic capillary junction tightening and lymphangiogenesis, occurs after VACV s.s., but not VACV i.d., resulting in reduced fluid transport [36]. This mechanism of lymphatic remodeling appears to require active viral replication and wounding and is dependent upon VEGFR2 signaling to regulate fluid transport [37]. After subcutaneous (s.c.) injection in the footpad, multiple viruses including vaccinia (VACV), modified Vaccinia Ankara (MVA), and Zika can enter LN conduits and are detected in the dLN within minutes, and also infect paracortical DCs along the conduits as quickly as 1 h post-infection [17,38]. VACV prevents skin DC mobilization to the dLN, but the direct priming of T cells in the LN paracortex is detected within 6–12 h, indicating that VACV virions directly access lymphatic vessels after skin infection and infect LN-resident antigen-presenting cells [39,40].

After subcutaneous immunization, VACV infection results in the localization of infected DCs and subcapsular macrophages in the draining lymph node (dLN) [38]. Finally, recent work identified LN medullary cords as a niche to which preDCs are recruited, and in which locally available Flt3L accelerates cDC1 development to maintain local cDC1 abundance, further demonstrating an active role for lymphatics in antiviral immunity [41]. Adenoviral vectors expressing VACV viral proteins were used to test how keratinocyte-specific vs. more ubiquitously expressed viral proteins pose different requirements for DC uptake and lymphatic transport [42]. After intradermal (i.d.) vs. intravenous (i.v.) infection, VACV i.d. led to keratinocyte-specific peripherally expressed viral proteins that require cell-mediated trafficking and solicit lower CD8 T cell priming than those ubiquitously expressed after intravenous VACV, which disseminates passively and broadly via the lymphatic fluid [42]. 

### 2.3. Early Immune Evasion in Infected Keratinocytes

Despite limited VACV replication in keratinocytes, keratinocytes upregulate Th2 and immunoregulatory cytokines that include TGF-B, IL-10, and IL-13, which are associated with tolerance, while suppressing local Th1 and cytotoxic CD8+ T cell responses in the skin [28].

WR-VACV hijacks host cell machinery in infected primary mouse keratinocytes to promote the synthesis of specific viral proteins, such as E3, encoded by gene E3L, that can dampen skin inflammatory responses. The expression of protein E3 suppresses proinflammatory cytokine production, including IFN-B, IL-6, CCL4, and CCL5, through the inhibition of PKR and NF-κB pathways. Additionally, E3 hinders the keratinocyte activation of IRF3 and NF-κB via a mitochondrial antiviral signaling protein-dependent pathway (MAVS), which functions as an adaptor for cytoplasmic viral RNA sensors RIG-I and MDA5 [43]. 

In individuals with abnormal keratinocyte function, such as those with pre-existing conditions like atopic dermatitis and acne, vaccinia infection may lead to uncontrolled viral dissemination [44]. VACV vaccination for smallpox may induce hyper-IgE syndrome due to STAT3 gene mutations, resulting in chronic eczema-like conditions [45]. In normal and transformed cells, STAT3 plays a host-protective role, promoting protein transcription that enhances cell cycle progression and antagonizes apoptosis [46]. It has been further shown that STAT3 plays a crucial function in keratinocyte defense against the smallpox vaccine ACAM2000™ through rapid programmed keratinocyte necrosis, contributing to the anti-vaccinia defense of the skin [44]. A topical STAT3 inhibitor (Stattic) led to higher viral titers and more severe infection in mice that underwent scarification with ACAM2000™, indicating that STAT3 inhibition impairs mechanisms by which keratinocytes control VACV infection [44]. 

### 2.4. Cell Death

VACV has strain-specific immune evasion mechanisms that inhibit cell death in host cells to prolong the survival of host cells, facilitating extended viral replication and propagation, ultimately leading to increased viral production [47,48]. In particular, by preventing apoptosis, VACV evades immune surveillance by phagocytic cells, including APCs, which recognize vesicles of apoptotic cells called apoptotic bodies, thereby initiating immune responses via danger signaling [48,49]. 

VACV proteins F1 and N1 block apoptosis by inhibiting signaling in the apoptosome complex pathways [48,50]. Proteins B13 and B22 inhibit apoptosis induced by cell-intrinsic signals, including death receptor signaling through the Fas-associated protein with death domain (FADD) pathway via the inhibition of caspase-8 [46].

WR-VACV has additional mechanisms to modulate apoptosis in infected cells to evade the host immune response [51]. One key mechanism involves the gene expression of B13R, which encodes protein B13 that closely mimics cowpox virus cytokine response modifier A (crmA), with a high amino acid identity of 92% [52]. In THP-1 cell lines, crmA inhibits the IL-1B-converting enzyme (ICE), catalyzing the conversion of pro-1B to mature IL-1B, a potent cytokine released as part of the innate defense [53]. While the B13R gene product does not prevent systemic IL-1B production, it effectively inhibits apoptosis triggered by anti-FAS antibody or TNF in combination with cycloheximide in the infected host cell [54]. 

VACV Copenhagen lacks the expression of functional B13, but is able to prevent apoptosis by interfering with the mitochondrial apoptotic cascade via the modulation of the permeability transition pore (PTP) complex, even when cells are treated with compounds that typically induce PTP opening, such as atractyloside and t-butylhydroperoxide [55]. Though the exact viral protein responsible for this mechanism in VACV Copenhagen has not yet been identified, this modulation of the apoptotic pathway not only aids in the virus’s survival within the host cells, but contributes to the evasion of immune surveillance mechanisms. By preventing apoptosis through various mechanisms via various viral proteins, VACV strains are thus able to effectively evade the immune response and establish a favorable environment for viral replication and spread. 

### 2.5. Generation of Pro-Inflammatory Homologs 

VACV employs other sophisticated strategies to undermine the host immune system. Tumor necrosis factor-alpha (TNF-α) is a potent inflammatory cytokine crucial for modulating the interplay between innate and adaptive immune responses [56] and promotes leukocyte recruitment to the infection site [57] through its receptors TNFR1 and TNFR2. The most well-characterized anti-TNF-α strategy employed by VACV is encoding multiple TNF receptor 1 homologs (vTNFR1), which effectively bind and sequester TNF-α, subsequently impeding its function within resident skin cells [56,58]. Moreover, mice deficient in TNFR1 exhibited compromised immune responses to cutaneous VACV infection, manifesting a phenotype resembling eczema vaccinatum with heightened IFNg production and CD8+ T cells in spleens and dLNs. TNFR1 deficiency is a skin-specific immune impairment because TNFR1 is exclusively expressed in keratinocytes, the primary cells infected during VACV vaccination in humans via skin scarification [56]. 

### 2.6. Interferon and Other Immune Responses

VACV enters the host cell through membrane fusion or endocytosis, and the recognition of viral components by pattern recognition receptors (PRRs) activates the innate immune response. These PRRs include Toll-like receptors (TLR2 and TLR9), cytosolic sensors such as RIG-1-like receptors (RLRs) and NOD-like receptors, and DNA sensors such as cGAS [59,60,61,62,63]. PRR signaling activates downstream signaling pathways, inducing the activation and translocation of transcription factors such as IRF-3 (Interferon Regulatory Factor 3) and NF-κB (Nuclear Factor-kappa B) to the nucleus of the host cell and upregulating the gene expression of proteins involved in type I interferon production (IFNa and IFNb). Type I interferons bind to the cell surfaces of autocrine and paracrine cells, initiating a signaling cascade via the JAK-STAT pathway to transcribe various interferon-stimulated genes (ISGs) that inhibit viral replication, induce apoptosis in infected cells, and enhance immune responses. During infection with VACV, type I and II interferon signaling are thought to play non-redundant roles, with the type I interferon response more crucial in the initial control of viral spread and type II playing a role in the activation of the adaptive response [64]. VACV evades host immunity by interfering with several steps of this pathway, as described below [65].

### 2.7. VACV Immunomodulatory Proteins 

As VACV enters the host cell through a membrane fusion process, the viral core and two flanking protein structures (lateral bodies) are released into the cytosol [66]. Though these lateral bodies are not entirely characterized, VACV phosphatase H1 (VH1), an immunomodulatory protein, is contained in VACV lateral bodies (and has an equivalent in VARV) and is released in a proteasome-dependent manner after VACV host cell entry [66]. VH1 inhibits the phosphorylation of transcription factors that play a critical role in type I and type II interferon signaling, STAT1 and STAT2, thus preventing interferon-stimulated immune responses [67]. 

Poxvirus immunomodulatory proteins such A52, B15, and K7 target the NF-κB pathway to inhibit the expression of proinflammatory cytokines and chemokines, thus evading immune control. A52 and K7 obstruct IL-1R-associated kinases (IRAK) and the activation of TNF receptor-associated factor 6 (TRAF6) [68,69], while B15 inhibits IkBa phosphorylation and proteasome degradation [70], halting the nuclear localization of NF-κB. NYVAC (generated from the Copenhagen strain by the deletion of 18 viral open reading frames [71]) infection restrains NF-κB binding to the κB site and diminishes the transcription of proinflammatory signals that recruit immune cells essential in the innate immune response [72]. This limits the generation of the first line of cellular defense comprising monocytes, DCs, natural killer (NK) cells, neutrophils, and B cells, impeding the transportation of VACV antigens to lymphoid organs and suppressing the initiation of direct antigen-specific T cell responses. The ablation of viral A52R, K7R, and B15R genes collectively expands the innate immune response, enhancing DC, NK, and neutrophil migration in addition to chemokine and cytokine production [73].

Furthermore, VACV protein F14 suppresses NF-κB-dependent inflammatory gene expression, diminishing the host’s ability to mount a robust antiviral response. Although VACV encodes 15 other proteins downstream of host receptor signaling that inhibit NF- κB activation, a VACV strain without these proteins still manages to suppress NF- κB activation. Using co-immunoprecipitation assays, F14 protein was identified to interact with CREB-binding protein (CBP), a co-activator of NF-κB, disrupting interactions between p65 and CBP. In mice, the intradermal immunization of recombinant VACV lacking F14 in the ear pinnae resulted in diminished lesion size and lower viral titers due to a functioning NF-κB pathway that stimulated an effective antiviral response [74]. 

VACV also evades the host immune response by inhibiting DC function. This evasion strategy includes blocking cytokine signals through the expression of decoy receptors for interferons, interleukin (IL)-1b, and TNF-α [75,76]. Additionally, VACV protein A52R interferes with TLR complex formation, which recognizes VACV antigen and DNA, thus disrupting the pathways associated with DC maturation. Notably, a VACV mutant lacking A52R demonstrated reduced virulence compared to the wildtype (WT) when used to intranasally infect mice [70]. VACV infection has further been observed to induce apoptotic cell death and block the maturation of human DCs [77]. This multifaceted interference with DC functions highlights the sophisticated mechanisms employed by VACV to undermine the host immune response. 

### 2.8. Immune Evasion by Other VACV Strains

The intradermal administration of WR-VACV has been observed to diminish the expression of the gene encoding virulence factor N1. In addition to its anti-apoptotic capabilities, N1, a glycosylated non-covalent homodimer predominantly found within infected cells, acts as a virulence factor by inhibiting the activation of IRF3 and NF- κB [50]. The deletion of N1L (and subsequent lack of immune suppression) diminishes VACV virulence in intranasal and intradermal models, confining infection locally post-intradermal immunization and limiting dissemination to other organs. Because the infection is contained locally in the dermis, with reduced lesion size and diminished viral titers in infected ears in the absence of N1L, it supports the notion that cutaneous VACV infection may prompt a dermal-specific immune evasion mechanism [78]. 

MVA is marked by a loss of 31 kB from its genome, resulting in diminished replicative capacity in human cells [79,80]. However, one of the critical genes that is not lost during the attenuation process is the E3L gene, encoding for E3 protein, which inhibits the host interferon response through blocking the activation of IRF3 and IRF7, both transcription factors necessary for IFNa/b response [81,82]. MVA-ΔE3L is not able to grow in CEF, and reinsertion of E3L rescues productive replication in CEF [83]. As a candidate vaccine vector for a potential method of antigen delivery, it is important to note that that viral late transcription and late protein biosynthesis is impeded in MVA-ΔE3L-infected HeLa cells [84,85].

Despite weakened virulence, MVA remains proficient at antagonizing the innate immune response by downregulating NK and T cell ligands [86]. Furthermore, MVA influences host factors by regulating viral gene expression, particularly during the late stages of infection, to control components of the nuclear pore complex (NPC) crucial for poxvirus replication [86]. Upon cellular detection of MVA, an upsurge in host antiviral responses ensues, with elevated innate-immunity-associated proteins, including interferon-stimulated genes (ISG20) and viral DNA sensor IFI16. MVA strategically counters host antiviral defenses by downregulating factors such as metallopeptidase inhibitor 2 (TIMP2), transforming growth factor B-1 (TGFB-1), and antiviral restriction factors like TRIM5. MVA’s expression of C6 viral protein targets TRIM5, prompting its proteasomal degradation and subverting its antiviral function [86]. 

## 3. Vaccinia and Adaptive Immunity

### 3.1. Early Cellular Events in Generating Adaptive Immunity

During and after the early innate immune events mentioned above, VACV establishes a long-term humoral and cellular protective immune response in both human and murine hosts. Access to professional antigen-presenting cells (APCs) including DCs is critical for robust immune responses triggered by VACV, activating both CD4+ and CD8+ T lymphocytes [38,87,88]. In the skin, DCs exist as immature cells and perform antigen uptake and processing [89,90,91,92]. Following antigen acquisition, skin DCs migrate to the dLN and undergo maturation, exhibiting increased expression of costimulatory and HLA molecules essential for T cell activation [93,94]. The consequent CD8+ T cell response significantly contributes to both viral clearance and protective immunity [35]. Both CD8+ T cells and CD4+ T cells contribute to early lytic clearance of VACV-infected APCs, although the contribution of other innate cells such as NKs is not yet well characterized [38]. Dozens of VACV antigen epitopes have been found after both mouse and human vaccination [95]. 

#### 3.1.1. DC Maturation and Antigen Presentation 

In addition to viral tropism, antigen presentation and DC kinetics also play a role in the immunogenicity of VACV antigens. Immature DCs use endocytic mechanisms to sample and recognize pathogens and damage, and in this case VACV antigen through PRRS, TLRs, RLRs, NLRs, CLRs (c-type-lectin receptors), cytokine and chemokine receptors, and DNA sensors [59,60,61,62,63,96,97]. Upon recognition, DCs migrate to secondary lymphoid organs and mature, upregulating MHC and T cell costimulatory molecules to present antigen to T cells [90,98]. CD8+ T cell activation depends upon a) the direct presentation of endogenous proteins by infected cells on MHCI, and b) the cross-presentation of phagocytosed apoptotic and necrotic cells by dendritic cells on MHCI [99,100].

In vitro, immature bone-marrow-derived dendritic cells (BMDCs) infected by VACV cannot mature—an essential prerequisite to elicit VACV-specific T cell activation by direct presentation—and often undergo apoptosis and secondary necrosis [77,101]. VACV blocks DC maturation through many mechanisms, including by encoding receptor homologs of IL-1β, TNF-α, IFN-α, and IFN-β to inhibit interferon signaling [77]. Vaccinia blocks the ex vivo maturation of immature murine and human DCs upon infection, preventing the direct presentation of viral antigen and DC migration, as well as upregulation of costimulatory and maturation proteins including CD86 and HLA-DR [77]. However, upon ex vivo infection with VACV, previously matured murine and human DCs retain expression of costimulatory markers and directly present VACV antigen for T cell activation [101].

In contrast to directly infected DCs whose maturation is suppressed, uninfected DCs exposed to dying cells and local licensing and inflammatory cues have higher levels of MHCI expression and costimulatory molecules, as well as IFN-β production, which might imply an enhanced ability to generate a vaccinia-specific T cell response [102]. However, MHCII expression is simultaneously downregulated, thus inhibiting antigen presentation to CD4+ T cells and dampening host immunity [102]. 

Cross-presentation is a potent mechanism of T cell activation by which DCs phagocytose pathogens, dead cells, and particulate, and present exogenous antigens on MHCI class I molecules via the TAP1-TAP2 pathway [87,103,104]. DC1s are specialized for cellular death sensing and cross-presentation, and migratory DCs have also been shown to be capable of cross-presentation [103,105]. Although monocytes can phagocytose cells, they are markedly less proficient in cross-presenting VACV antigens [106]. Antigens identified as T cell epitopes in both human infection and in vivo mouse studies are expressed largely under early VACV promoters and are frequently identified as virulence factors or proteins associated with viral genome regulation [95]. As mentioned above, because VACV cannot replicate in myeloid cell populations but can impair DC maturation, both endogenous and model antigens driven by late viral promoters are selectively transcribed and less able to drive CD8+ T cell immunogenicity than proteins expressed early during viral infection [96]. However, some late VACV-promoter-driven antigens can be cross-presented by uninfected DCs and directly presented by infected DCs [95].

The dependence upon direct presentation in driving VACV-specific immunity depends upon the route of VACV administration. This was tested by using recombinant vaccinia expressing US11, a human cytomegalovirus endoplasmic reticulum (ER)-resident membrane protein that targets endogenous class I heavy chains in the ER for cytoplasmic degradation, preventing the direct loading of antigens on MHC class I in infected APCs [107]. Immunization by intraperitoneal injection (i.p.) and intravenous injection (i.v.) depend more upon direct priming by infected mature DCs for the generation of cytotoxic T lymphocytes (CTLs), while subcutaneous (s.c.) and intradermal (i.d.) VACV infections can solicit antigen-specific CD8+ CTL immunity independent of direct presentation [108]. Hence, the route of immunization appears to shape both early viral clearance and late protection, including clearance kinetics and the mode of DC antigen presentation. 

#### 3.1.2. DC Subsets and Their Role in Vaccinia and Skin Infection

DCs are critical to the generation of a vaccinia-virus-specific T cell response [102,108]. Conventional DC1s (cDC1s) dependent upon transcription factor *Batf3* are adapted to detecting dead and dying VACV-infected cells by the expression of the C-type lectin domain family 9 member A (DNGR-1) [109]. LN XCR1^+^-expressing Batf3^+^-dependent DCs correspond to the skin migratory CD103+ CD11b− DC equivalent and are most capable at the cross-presentation of the exogenous Ova antigen injected intravenously [110].

DNGR-1 is primarily expressed upon cDC1s and binds F-actin, an intracellular cell component that is exposed following membrane rupture upon apoptotic and necrotic cell death that occurs during vaccinia infection [109]. DNGR-1 signals through kinase SYK and phosphatase SHP-1 to inflammatory chemokine production upon tissue damage [109]. Importantly, DNGR-1 promotes endocytic or phagocytic compartment rupture and directs dead-cell-associated antigens into a recycling endosomal compartment for endogenous MHC class I processing to favor cross-presentation and generate an anti-VACV CD8+ T cell response [111]. Using mice deficient in DNGR-1 (*Clec9a^gfp/GFP^*) or DC1 (*Batf3^−/−^*), a crucial role was identified for DC1s in cross-priming VACV-specific CD8+ T cells and generating tissue-resident memory CD8+ T cells (T_RM_) against VACV, including through the production of IL-12, IL-15, and CD24 expression [112]. The role of cDC1 was also tested through their expression of Langerin, using Langerin^DTR^ mice, in which diphtheria toxin administration singularly or repeatedly allowed for Langerhans cell depletion or both Langerhans cell and Langerin+ DC depletion, respectively [113]. Upon the depletion of Langerin+ DCs, the antigen-specific CD8+ T cell effector function was reduced by approximately 50%, but was not absent after VACV skin scarification, demonstrating a likely role for other antigen-presenting populations. DC subset sorting and assessment of antigen presentation ex vivo revealed that CD207+ (Langerin) CD103+ dermal DCs cross-present antigens to CD8+ T cells after recombinant VACV-OVA [114] immunization; however, not all populations of tissue migratory DCs were similarly assessed, such as the 25–40% that are Langerin-low and CD11b-low and associated with allergy and contact sensitivity [105,115]. 

The role of other DCs in vaccinia protection has yet to be tested. The expansion of MHC-II^+^ CD11c^hi^ CD11b^hi^ CD64 macrophages in VACV-infected dLN [42,116] has also been observed. Lymphoid-resident cDC2 and dermal migratory cDC2 have a common origin from FLT3L (Fms like tyrosine kinase 3 ligand)-responsive conventional DC progenitors, but only the dermal migratory cDC2 subset expresses CD301b and actively promotes IL-17-mediated psoriasiform dermatitis [117,118,119]. Dermal CD301b+ cDC2 was also shown to produce IL-23, which induces the dermal gamma delta T cell production of protective IL-17 during *Candida albicans* infection [120]. Dermal CD301b+ cDC2s are distinct from epidermal or CD207+ (Langerin) dermal DC1s, are dependent upon transcription factor interferon regulatory factor 4 (IRF4), and are significant drivers of Th2 cell development and immunity upon infection with Nippostrongylus brasiliensis [121,122]. Topical antigen can be acquired and presented by Langerhans cells, and dermal CD11b+ cDC2s can uptake and present topical antigen to CD4+ T cells. However, only dermal IRF-4-dependent CD11b+ cDC2s were observed to be both required and sufficient to present topically-derived antigen in the dLN leading to T regulatory cell induction [123]. A respiratory infection model of the single-stranded RNA pneumonia virus of mice (PVM) was used to characterize the ability of inflammatory cDC2s to induce antigen-specific T cell responses [124]. Due to inflammation in the lung, Toll-like receptor signaling, and type I interferon signaling, the proportion of both monocyte-derived cells and inflammatory cDC2s significantly increased in the dLN at 8 days post-infection, when the viral load was highest [124]. However, only inflammatory cDC2s excelled at inducing antigen-specific CD4+ T cell proliferation and IFN-γ production, while cDC1s induced antigen-specific CD8+ T cell proliferation. Despite this, inflammatory cDC2s have been able to drive antigen-specific CD8+ T cell proliferation at a higher rate than classical cDC2s [124]. 

### 3.2. DC Maturation Antigen Processing and Presentation in MVA

Unlike wildtype VACV, MVA induces both IFN-α and IFN-β production in cDCs, but not pDCs [125,126]. After modified Vaccinia Ankara (MVA) infection, DC maturation and downstream antigen processing and presentation are critically dependent upon stimulator of interferon genes (STING) signaling due to the requirement for DNA sensor cGAS and subsequent type I interferon response [127]. After vaccination with MVA expressing various model antigens, both the quantity and quality of the CD8+ T cell response (based upon the expression of CD107a, IFN-γ, and MIP-1α) was downregulated in STING knockout mice, although the memory CD8+ T cell response was unaffected [128]. Ex vivo experiments showed that STING signaling is relevant in directly infected immature DCs to activate mature phagocytes for cross-presentation, which is necessary in bystander DCs for maturation and necessary in cross-presenting DCs for antigen processing and presentation. Finally, in the absence of STING, both IFN-α and IFN-β responses in BMDCs infected in vitro were completely lost, and in vivo STING KO mice infected with MVA had abrogated T cell recruitment chemokine and pro-inflammatory cytokine production. 

Plasmacytoid DCs (pDCs) depend on CXCR3 to migrate to the sites of infected macrophages, positioning them at the infected sites [129]. They also depend on CCR5 to migrate to clusters of antigen-specific CD8+ T cells after infection with MVA by subcutaneous delivery in the footpad [129]. There, pDCs serve as a critical source of type IFN, which induces the expression of CD40, CD80, and CD86 on XCR1+ DCs recruited to the site of CD8+ T cell priming by XCL1 secretion [130]. In turn, these XCR1+ DCs are potent in the cross-presentation of viral antigens to CD8+ T cells, and are crucial in non-replicative MVA infection [129,130]. This pDC, CD8+ T cell, and XCR1+ DC axis thus requires spatial and functional cooperativity in order to drive optimal CD8+ T cell priming upon viral infection [130]. 

### 3.3. Late Protection: CD8 T Cell Memory in Vaccinia

Naïve CD8+ T cells receive “signal one” from the T cell receptor (TCR) recognizing the antigen presented on MHCI, and “signal 2” through co-stimulation by ligation of CD40L expressed on T cells binding CD40 on the same antigen-presenting cell (APC) [131,132,133,134,135,136]. Inflammatory cytokines including IL-12 and interferon α/β then provide “signal 3” to generate effector and memory populations [137,138]. CD8+ T cells are crucial for long-term protective immunity to tumors, vaccines, and viruses, and differentiate into T circulating memory (T_CM_), T effector memory (T_EM_), and tissue-resident memory cells (T_RM_). Vaccination with recombinant VACV_SS_ induces a superior T-cell-mediated immune response over intramuscular (i.m.) or subcutaneous (s.c.) modes by generating protective T_CM_ and tissue-resident T effector memory (now known as T_RM_) cells against secondary challenge [35]. Administration of FTY720, a sphingosine-1-phosphate antagonist-blocking T_CM_ LN egress, demonstrated that both VACV-specific T_RM_ and T_CM_ are generated after VACV_SS_, while only T_RM_ are needed to clear virus after a secondary cutaneous skin viral challenge, although both T_RM_ and T_CM_ are needed to provide complete protection against lethal secondary intranasal challenge [34]. Activated T cells, following localized viral infection and priming in dLNs, acquire additional tissue-homing imprinting programs including the expression of CCR4, E-selectin, and P-selectin ligands independently of further antigen presentation, with the initial homing imprint persisting as the predominant memory phenotype [35]. Immunodominance, known as a targeted immune response to only a few antigenic peptides, is heightened following vaccinia administration through immunization in the periphery (i.d. and s.c.) compared with routes preceding systemic viral dissemination (i.p. and i.v.) [139]. Cutaneous infection generates both CD8+ T circulating memory cells (T_CM_) and CD8+ T_RM_ in peripheral tissues, whereas intraperitoneal (i.p.) injection does not generate T_RM_ [140]. Both T_CM_ and T_RM_ are adequate for anti-tumor immunity, with T_CM_ retaining the potential to generate T_RM_, though T_RM_ improve anti-tumor efficacy upon secondary challenge [140]. Batf3 cross-presenting DCs are required to generate T_RM_, as well as to reactivate the circulating memory tumor response, and the use of FTY720 demonstrated that T_RM_ were sufficient for anti-tumoral secondary protection [140]. 

T_RM_ established from shared progenitors with circulating memory populations [141] seed the tissue broadly, but are concentrated at sites of original infection. After intravenous priming with peptide Ag-coated DCs and secondary challenge with either skin infection, subcutaneous infection, or local inflammation, both local inflammation and antigen recognition were found to successfully recruit T_RM_ in the skin for accumulation and retention, regardless of the original priming site [142]. Modified Vaccinia Ankara generates lung and skin T_RM_ after immunization via skin scarification. Notably, the skin T_RM_ in mice treated with MVA skin scar (MVA_SS_) had a different transcriptional profile and were more abundant compared to T_RM_ in mice immunized by i.d., s.c., and i.m. routes, though the transcriptional profiles after various skin immunization methods were more related than that after i.m. infection. Upon lethal rechallenge in μMT mice that, due to a mutation in the heavy (μ) chain of IgM, cannot produce mature B cells and cannot generate antibodies, s.s. and intratracheal (i.t.) immunization both generated sufficient T_RM_ in skin and lung, and were able to protect against lethal respiratory challenge, whereas intraperitoneal immunization did not [143]. Therefore, the route of immunization again changes the quality of the immune response according to site specificity. 

#### 3.3.1. CD4 T Cell Help

CD4+ T cells contribute to CD8+ T cell expansion and memory development in many contexts [144,145]. Prior studies determined that CD8+ T effector function, after some inflammatory infections, do not require CD4+ T cell help during the initial antigen encounter and pathogen clearance, but require CD4+ T cell help in the generation and maintenance of CD8+ T memory cells. For example, in experiments testing either infection with Listeria monocytogenes expressing the LCMV GP(33–41) epitope or LCMV infection, a reduction in CD8+ memory cell number and function, but not the primary expansion of effector cells, was observed in CD4-deficient mice compared to WT mice [146]. The memory CD8+ T cells primed in CD4-deficient or depleted mice during priming had worse recall response, including IFN-γ production, proliferative capacity, and cytotoxic activity [146]. The requirement for CD4+ T cells in the initial priming stages of CD8+ T cell memory was postulated to be either that in the absence of an inflammatory infectious agent, CD4+ T cells are necessary to license APCs through CD40 signaling, or, alternatively, that CD8+ T cells experiencing persisting antigen exposure in a chronic infection require CD4+ T cell help for survival [146]. 

Route and CD4 help requirements: In a comparison of LCMV (i.v.), a virus-like particle infection expressing LCMV antigen gp33–41 (s.c.), and VACV engineered to express LCMV antigen gp33–41 (i.p.), the primary antigen-specific CD8+ T cell response for LCMV gp33–41 peptide was found to be independent of CD4+ T cell help, except in the case of i.p. vaccinia infection. Here, CD4+ T cell help was found to be indispensable for licensing DCs via CD40 to drive memory CD8+ T cell recall proliferation and survival [147]. Also, in MHCII-/- mice, VACV-specific CD8+ T cell generation was diminished only when VACV was administered via intraperitoneal infection, and not in intranasal infection, highlighting route-specific differences in immune requirements [148]. CD4 T cell-dependent IL-2/CD25 signaling was found to be the i.p. specific requirement needed to drive virus-specific CD8+ T cell expansion and survival [148]. However, without CD4+ T cell help, VACV-specific CD8+ T cells maintained similar effector function after both i.p and i.n. immunization [148]. This is because early VACV activation of CD4+ and CD8+ T cells occurs separately, with CD4+ T cells primarily associating with non-infected DCs and CD8+ T cells associating with infected DCs. In later infection stages, both T cell types translocate to the LN paracortex where they both interact with a distinct non-infected XCR1+ DC population [129]. In a VACV s.s. experiment, CD4-deficient or CD4+ T cell-depleted mice still experienced normal antigen-specific CD8+ T cell proliferation in the dLN. On the contrary, enhanced accumulation in the skin was observed [149]. Despite the differences in CD4 help in these model contexts in vaccinated individuals, CD4 help may be important, with CD4 persistence of up to 55 years after infection [150].

#### 3.3.2. Late Protection: B Cells 

The establishment of long-term humoral immunity after vaccination relies on the coordinated efforts of plasma cells with a long lifespan, and memory B cells [151]. Long-lived plasma cells maintain serum antibody levels, and memory B cells swiftly trigger the antibody response upon re-exposure to antigens [152]. Memory B cells also replenish the reservoir of long-lived plasma cells, ensuring sustained antibody levels without pathogen re-exposure [153,154].

While the focus on B cell humoral immunity has been limited post-infection with VACV in mice, B cells are considered a strong measure of immunity in humans. In fact, memory B cells specific to the smallpox vaccine post-Dryvax^®^ vaccination have been detected over 50 years after vaccination in immunized individuals at only 10-fold lower levels than at peak levels [155]. The presence of anti-VACV antibodies was confirmed in the sera of 35/38 vaccinated individuals [155]. An enzyme-linked immunosorbent assay (ELISA) detected neutralizing antibodies in serum samples from post-immunization time points up to 59 years later [155]. Further, over the course of 25–75 years, more than 90% of individuals vaccinated with VACV against smallpox maintained anti-VACV humoral or cellular immunity. While the VACV-specific T cell responses declined over 8–15 years, the VACV-specific antibody responses were stable over 75 years post-vaccination [156]. The longevity of antibody presence demonstrates the effectiveness of VACV as the smallpox vaccine in providing long-lasting immunity and underscores that long-lasting antibodies can be a measure of VACV vaccine efficacy. 

In the context of recombinant VACV immunization, mice exhibit effective peripheral and central memory T cell response upon WR-VACV secondary challenge, attributed to the presence of tissue-resident memory T cells (T_RM_) generated by the original skin scar immunization [34]. To explore the requirement for B cells upon secondary challenge, uMT mice lacking mature B cells were immunized via the s.s. route and showed complete protective immunity against lethal WR-VACV challenge (i.n), even in the absence of neutralizing antibodies [34]. As skin-scar-immunized uMT memory mice lost protection upon T cell depletion, the results implicate B cells are less critical for protection during secondary respiratory challenge [34]. 

### 3.4. In WR-VACV

Studies in Rag.1−/− mice, which lack functional B cells and CD4+ T cells, reveal their inability to control WR-VACV viral spread, underscoring the indispensable role of these cells in combating viruses. The depletion of CD8+ T cells in MHC class II−/− exacerbate disease severity, although they marginally contribute the primary protection against VACV [157]. In the mice depleted of CD4+ T cells, MHC class II−/− and IgH−/− mice (lacking B cell responses), WR-VACV is unable to be cleared, which is attributable to their defect in initiating IgG and IgM response [157]. 

In individuals immunized with WR-VACV, VACV-specific memory B cells persist for over five decades, constituting 0.1% of circulating IgG+ B cells [155]. The memory B cell response exhibits a biphasic pattern, initially declining and then stabilizing over decades. This stabilization could stem from the migration of circulating memory cells, the programmed cell death of B cells to be replenished, or the acute retention of antigens by DCs. This mechanism precedes a reduction in memory B cell numbers, reaching antigen-independent equilibrated levels [155]. 

## 4. Concluding Remarks 

Vaccinia immunization has historical significance in eradicating smallpox, and emerging outbreaks like the monkeypox outbreak have highlighted vaccinia’s continued relevance in vaccine development and therapeutic interventions. Vaccinia remains a fascinating subject of study for host–pathogen defense as the vaccination route, innate defense mechanisms, modes of viral immune evasion, strain-specific differences, lymphatic drainage, and host immunity collectively shape outcomes. 

Despite a wealth of knowledge identifying principles governing long-term protective successful vaccinia immunity, there remains a critical gap in informing future vaccine design and delivery. Vaccinia employs a range of evasion and inhibition strategies against the host’s immune response, allowing for successful propagation within the host. Further, DCs play a critical role in the generation of adaptive immunity to VACV, orchestrating T lymphocyte activation, and in the establishment of long-term protective immunity. The role of DCs other than cross-presenting DC1s in vaccinia immunization has not been thoroughly elucidated, nor have the specific mechanisms for s.s. superiority over other routes, including i.d., in recruiting skin Trm accumulation and retention been entirely defined. Furthermore, skin-specific immune mechanisms, including the keratinocyte production of inflammatory and tolerogenic cytokines, and the contribution of CD4+ T cell–DC interactions specifically in VACV s.s. have not been interrogated in comparison to other routes of vaccination and in terms of long-term protective immunity. Understanding the mechanisms of host immune evasion, the interactions between vaccinia and DC subsets, route-specific differences in immune requirements, and the dynamics of CD8+ T memory cells, CD4+ T cells, and B cells in establishing long-term immunity is critical for the future development of effective immunotherapies and vaccines utilizing poxviruses.
