# Correction: Hsu et al. Vaccinia Virus: Mechanisms Supporting Immune Evasion and Successful Long-Term Protective Immunity. *Viruses* 2024, *16*, 870

**DOI:** 10.3390/v18040401

**Published:** 2026-03-24

**Authors:** Joy Hsu, Suyon Kim, Niroshana Anandasabapathy

**Affiliations:** 1Weill Cornell Graduate School of Medical Sciences, New York, NY 10065, USA; 2Department of Dermatology, Weill Cornell Medicine, New York, NY 10021, USA; 3Department of Microbiology and Immunology, Weill Cornell Medicine, New York, NY 10021, USA; 4Meyer Cancer Center, Weill Cornell Medicine, New York, NY 10021, USA; 5Englander Institute of Precision Medicine, Weill Cornell Medicine, New York, NY 10021, USA; 6Renaissance School of Medicine, Stony Brook University, Stony Brook, NY 11794, USA; suyon.kim@stonybrookmedicine.edu

The email address of the corresponding author has been updated. This update does not affect the scientific content of the article

## Missing Citation

Due to an error during the production process, the reference list was published incorrectly. The corrected references have now been updated in the article. In the original publication [1], “Mann, B.A.; Huang, J.H.; Li, P.; Chang, H.C.; Slee, R.B.; O’Sullivan, A.; Anita, M.; Yeh, N.; Klemsz, M.J.; Brutkiewicz, R.R.; et al. Vaccinia virus blocks Stat1-dependent and Stat1-independent gene expression induced by type I and type II interferons. *J. Interferon Cytokine Res.* **2008**, *28*, 367–380. https://doi.org/10.1089/jir.2007.0113.” was not cited. The citation has now been inserted in Section 2, Vaccinia and Innate Immunity, Section 2.7. VACV Immunomodulatory Proteins, paragraph 1, number 67 in the reference list, and it should read:

As VACV enters the host cell through a membrane fusion process, the viral core and two flanking protein structures (lateral bodies) are released into the cytosol [66]. Though these lateral bodies are not entirely characterized, VACV phosphatase H1 (VH1), an immunomodulatory protein, is contained in VACV lateral bodies (and has an equivalent in VARV) and is released in a proteasome-dependent manner after VACV host cell entry [66]. VH1 inhibits the phosphorylation of transcription factors that play a critical role in type I and type II interferon signaling, STAT1 and STAT2, thus preventing interferon-stimulated immune responses [67].

With this correction, the order of some references has been adjusted accordingly. 

## Text Correction

There was an error in the original publication. Cytomegalovirus was incorrectly spelled cytolomegavirus. A correction has been made to Section 3, Vaccinia and Adaptive Immunity, Section 3.1.1. DC Maturation and Antigen Presentation, paragraph 5:

This was tested by using recombinant vaccinia expressing US11, a human cytomegalovirus endoplasmic reticulum (ER)-resident membrane protein that targets endogenous class I heavy chains in the ER for cytoplasmic degradation, preventing the direct loading of antigens on MHC class I in infected APCs [107].

## References

With this correction, the order of reference citations from reference 43 onwards has been adjusted accordingly.

The authors state that the scientific conclusions are unaffected. This correction was approved by the Academic Editor. The original publication has also been updated.
